# Author Correction: All semiconductor enhanced high-harmonic generation from a single nanostructured cone

**DOI:** 10.1038/s41598-020-63113-5

**Published:** 2020-04-07

**Authors:** Dominik Franz, Shatha Kaassamani, David Gauthier, Rana Nicolas, Maria Kholodtsova, Ludovic Douillard, Jean-Thomas Gomes, Laure Lavoute, Dmitry Gaponov, Nicolas Ducros, Sebastien Février, Jens Biegert, Liping Shi, Milutin Kovacev, Willem Boutu, Hamed Merdji

**Affiliations:** 1 LIDYL, CEA, CNRS, Université Paris-Saclay, CEA Saclay, Saclay, 91191 Gif sur Yvette France; 2 SPEC, CEA, CNRS, Université Paris-Saclay, CEA Saclay, Saclay, 91191 Gif sur Yvette France; 3 Novae, ZA du Moulin Cheyroux, Moulin, 87700 Aixe-sur-Vienne France; 4 University Limoges, CNRS, XLIM, UMR 7252, 87000 Limoges, France; 5 ICFO–The Institute of Photonic Sciences, Mediterranean Technology Park, Av. Carl Friedrich Gauss 3, 08860 Castelldefels, Spain; 6 Leibniz Universität Hannover, Institut für Quantenoptik, Welfengarten 1, D-30167 Hannover, Germany

Correction to: *Scientific Reports* 10.1038/s41598-019-41642-y, published online 05 April 2019

The Acknowledgements section in this Article is incomplete.

“We acknowledge Franck Fortuna and Laurent Delbecq for access and support to the nano-focused ion beam at the CSNSM laboratory (IN2P3, Paris Saclay University). We acknowledge support from the PETACom FET Open H2020, support from the French ministry of research through the ANR grants 2014”IPEX”, 2016 “HELLIX”, 2016 “BISCOT”, 2017 “PACHA”, the DGA RAPID grant “SWIM” and from the C’NANO research program through the NanoscopiX grant, and the LABEX “PALM” (ANR-10-LABX-0039-PALM) through he grants”Plasmon-X”, “STAMPS” and “HILAC”. We acknowledge the financial support from the French ASTRE program through the “NanoLight” grant. We aknowledge support from Conseil Régional de Nouvelle-Aquitaine grant 2017 “FLOWA”. Financial support by the Deutsche Forschungsgemeinschaft, grant KO 3798/4-11 and from Lower Saxony through “Quanten- und Nanometrologie” (QUANOMET), project NanoPhotonik are acknowledged. J.B. acknowledges financial support from the Spanish Ministry of Economy and Competitiveness (MINECO), through the “Severo Ochoa” Programme for Centres of Excellence in R&D (SEV-2015- 0522) and the Fundació Cellex Barcelona.”

should read:

“We acknowledge Franck Fortuna and Laurent Delbecq for access and support to the nano-focused ion beam at the CSNSM laboratory (IN2P3, Paris Saclay University). We acknowledge support from the PETACom FET Open H2020, support from the French ministry of research through the ANR grants 2014”IPEX”, 2016 “HELLIX”, 2016 “BISCOT”, 2017 “PACHA”, the DGA RAPID grant “SWIM” and from the C’NANO research program through the NanoscopiX grant, and the LABEX “PALM” (ANR-10-LABX-0039-PALM) through the grants”Plasmon-X”, “STAMPS” and “HILAC”. We acknowledge the financial support from the French ASTRE program through the “NanoLight” grant. We acknowledge support from Conseil Régional de Nouvelle-Aquitaine grant 2017 “FLOWA”. Financial support by the Deutsche Forschungsgemeinschaft, grant KO 3798/4-11 and from Lower Saxony through “Quanten- und Nanometrologie” (QUANOMET), project NanoPhotonik are acknowledged. J.B. acknowledges financial support from the Spanish Ministry of Economy and Competitiveness (MINECO), through the “Severo Ochoa” Programme for Centres of Excellence in R&D (SEV-2015- 0522), the Fundació Cellex Barcelona, ERC Advanced Grant “TRANSFORMER”, Agreement No. 788218 and Laserlab-Europe (EU-H2020 654148).”

