# Supplementary material for: All semiconductor enhanced high-harmonic generation from a single nanostructured cone
Source: Sci Rep. 2019 Apr 5;9:5663. doi: 10.1038/s41598-019-41642-y (PMC6450872; doi:10.1038/s41598-019-41642-y)
Supplement: Supplementary file 1 — Supplementary information [file 41598_2019_41642_MOESM1_ESM.docx]

# Supplementary materials for

# All semiconductor enhanced high-harmonic generation from a single nanostructured cone

Dominik Franz^1^, Shatha Kaassamani^1^, David Gauthier^1^, Rana Nicolas^1^, Maria Kholodtsova^1^, Ludovic Douillard^2^, Jean-Thomas Gomes^3^, Laure Lavoute^3^, Dmitry Gaponov^3^, Nicolas Ducros^3^, Sebastien Fevrier^3,4^, Jens Biegert^5^, Liping Shi^6^, Milutin Kovacev^6^, Willem Boutu^1^ and Hamed Merdji^1*^

*^1^ LIDYL, CEA, CNRS, Université Paris-Saclay, CEA Saclay 91191 Gif sur Yvette France*

*^2^ SPEC, CEA, CNRS, Université Paris-Saclay, CEA Saclay 91191 Gif sur Yvette France*

*^3^ Novae, ZA du Moulin Cheyroux, 87700 Aixe-sur-Vienne, France*

*^4^ Univ. Limoges, CNRS, XLIM, UMR 7252, 87000 Limoges, France*

^5^ ICFO – The Institute of Photonic Sciences,

Mediterranean Technology Park, Av. Carl Friedrich Gauss 3, 08860 Castelldefels, Spain

^6^ Leibniz Universität Hannover, Institut für Quantenoptik, Welfengarten 1, D-30167 Hannover, Germany

**Movie S1. Dynamic enhancement of H5 and luminescence.** Data were taken at an intensity of 0.07 TW/cm^2^. The movie has been realized by assembling a transverse scan of the nanocone across the Gaussian beam at an integration time of 500 ms. A filter (FB420-10, Thorlabs) was used to select the spectral region around H5. The signal is monitored by using the same imaging setup shown in the experimental setup (Fig. 2). The first sequence of the movie shows the signal (H5 and luminescence) from the bare crystal. The nano-positioning system then moves the sample step by step (2 µm steps) and the CCD acquires images at each position, with the nanocone out of the laser beam focus at the beginning of the movie, centered in the focus of the beam at the middle of the movie and out of the focus at the end. All the images are then assembled and combined to a mp4-movie. The beam position is indicated by a whited dashed circle.


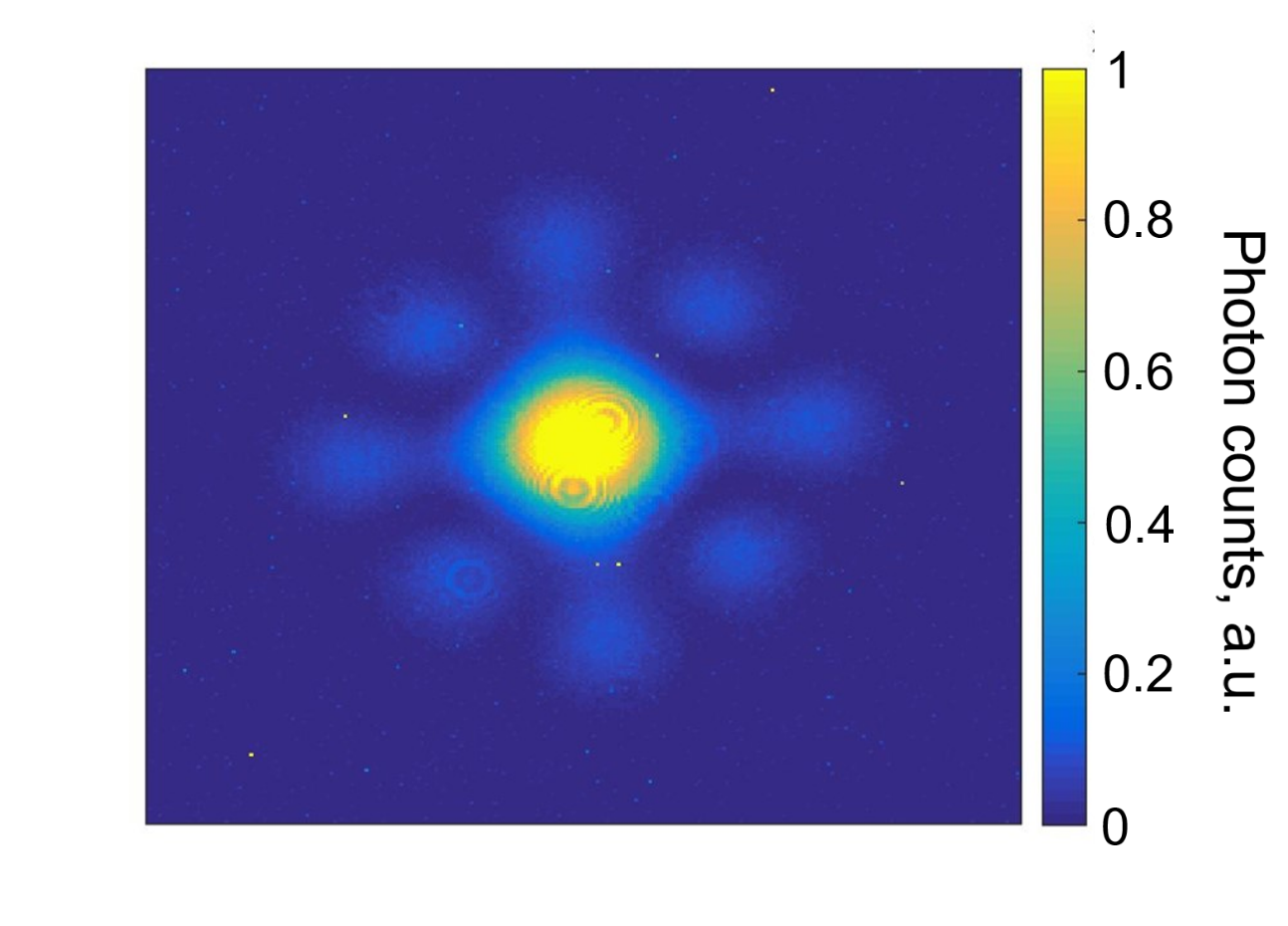


Figure S1:

Measured diffraction pattern shown in Fig. 5.
